# Predicting the natural history of artificial intelligence in travel medicine

**DOI:** 10.1093/jtm/taac113

**Published:** 2022-10-08

**Authors:** Gerard Thomas Flaherty, Watcharapong Piyaphanee

**Affiliations:** School of Medicine, University of Galway, Galway, Ireland; School of Medicine, International Medical University, Kuala Lumpur, Malaysia; Department of Clinical Tropical Medicine, Faculty of Tropical Medicine, Mahidol University, Bangkok, Thailand

**Keywords:** AI, machine learning, robotics, virtual reality, augmented reality

Research in various medical specialties has created excitement around the potential for artificial intelligence (AI) to transform clinical practice by improving the accuracy and efficiency of diagnosis and prognosis. Travel medicine is uniquely positioned to embrace its benefits. Education and training should reflect this and will require greater cooperation with data scientists.

The global public health response to the coronavirus disease 2019 (COVID-19) pandemic has accelerated the adoption of novel digital technologies in everyday life and in the workplace, particularly in the healthcare and education sectors.^1^ International travel has started to recover from the profound disruption caused by the pandemic. Aspects of the traveler experience have been permanently changed by the greater use of technology aimed at reducing the risk of transmission of severe acute respiratory syndrome coronavirus 2. The travel medicine community has demonstrated flexibility and resilience in adapting to the effects of the pandemic on routine clinical activity.^2^ The International Society of Travel Medicine (ISTM), through its COVID-19 Task Force, successfully navigated the pandemic by utilizing an immersive virtual reality platform to host its biennial conference in 2021 and pivoting to online delivery of its annual review course and certificate examination.^3^

Within the technology sector, AI has enabled the development of innovative systems that have been integrated into our everyday lives. These include intelligent web search algorithms, targeted advertising, entertainment content recommendations, product recommendations, smart watches that process inputs from accelerometer/biometrics sensors, and autonomous vehicles. AI has already found multiple applications in the travel industry but preventive travel medicine has been slow to embrace its potential. This editorial considers the current and future status of AI in travel and reflects on how travel medicine may benefit from its wider application in clinical practice and research.

The travel industry has been an early adopter of AI technology. Personalized travel recommendations based on travel patterns and preferences are a well-established feature of online trip searches. Flight price forecasting applications already alert travellers about favourable deals and the optimum time to make their travel bookings. Some providers employ chatbots to engage with the traveller. Social media sentiment analysis has multiple travel-related applications and may help the industry to improve health and safety aspects of their travel experiences. It is likely that personalized end-to-end travel planning will become the norm for travellers, with personalized recommendations based on the traveller’s health data. For example, package holidays may be customized to suit the traveller’s fitness levels and travellers with chronic medical conditions may be advised to travel to specific destinations with a high-quality medical infrastructure, linked with their travel insurance policies.

Facial recognition technology developed further during the COVID-19 pandemic and has already been deployed at major international airports. It is expected to gain greater acceptance among the traveling public as a touchless method of flight and hotel check-in, but its potential applications extend beyond this to user authentication when using unfamiliar computers overseas and as a means of ensuring traveler safety. Imagine a scenario where facial recognition of a traveler walking towards a terrorist attack, public demonstration or other disturbance triggers a smartphone notification with real-time safety advice. Opponents of such technology may not welcome such surveillance but, with proper safeguarding of personal data and civil liberties, the outcomes in terms of enhanced traveler safety and security may be significant. AI-assisted smart baggage handling may help to reduce the burden of lost or diverted passenger baggage. Robots are being used as disinfection devices at airports and as hotel concierges. A robot which transports vaccines from the refrigerator to the clinic room is already in use at the Travel Health Clinic at Mahidol Hospital for Tropical Diseases. Will robots eventually assist in the preparation and administration of vaccines? Robotic surgery is widely accepted now, particularly in the field of urology, so robotic travel health assistants may not stretch the imagination much further.

The volume of publications on AI in medicine has increased exponentially in recent years, especially in fields that rely on image-based or data-driven patient diagnosis, or where big data help to inform patient management or prognosis. Thus, AI research has featured prominently in highly visual specialties such as histopathology, haematology, radiology and dermatology with performance comparable to or better than human experts reported from studies of image analysis algorithms. Beyond the visual recognition domain, AI diagnostic skills are becoming more intelligent; witness IBM’s Watson diagnostic tools or Babylon Health, a chatbot offered by the UK’s National Health Service (NHS). AI has also found application in the analysis of genomic data in precision medicine and in novel drug discovery. It has been applied to infectious disease outbreak surveillance activities and to public health contact tracing during the COVID-19 pandemic.

A systematic review of patient and general public attitudes towards AI revealed that patients welcome the potential of AI to yield more accurate diagnoses, to support the training of physicians, to improve health access, to reduce healthcare costs by improving clinical workflow and to activate patients by encouraging them to seek health information or healthcare.^4^ The potential for AI to improve patient communication and to empower patients by improving their autonomy was also highlighted. Thus, it seems that, given its growing presence in our lives, AI applications in healthcare have received broad acceptance within the general population. Perceptions of healthcare professionals towards AI have also been largely positive. A study of fellows and trainees across various specialties in Australia and New Zealand found that 71% of doctors believed that AI would have a positive impact on their field of practice, with improved disease screening and less need for repetitive tasks recognized as distinct advantages of the technology.^5^ Medico-legal liability and the surrendering of some degree of control to the technology industry were identified as potential threats of AI.

As travel medicine practitioners, many of us are older than our specialty. We can anticipate exciting developments in our capacity for risk assessing and protecting travellers in the near future. It is therefore intriguing to reflect on the potential of AI for disruptive innovation in this field. AI is being used by our journals to capture insightful data by analyzing the public attention given to published articles. This journal was among the first to compare these altmetrics with conventional citation data.^6^ The potential application of AI in personalizing risk assessment and in prioritizing pre-travel health advice based on traveller characteristics, travel patterns and travel health records could be transformative. Will AI enable the travel health clinician to make accurate predictions about traveller behavior and risk avoidance strategies based on large volumes of training data from similar travelers? Will machine learning algorithms enable the identification of future last-minute travelers and provide an early warning system encouraging them to attend their pre-travel consultation earlier? The possibilities are endless.

There may be a role for virtual reality to assist in the preparation of anxious travelers or those embarking on emotionally or physically demanding trips abroad; indeed, the future carbon-conscious traveler may use immersive virtual reality platforms as a substitute for physical travel.^7^ Augmented reality may allow the traveler to use their smartphones to identify in real time potentially unsafe situations during travel (e.g. transportation options, violent public demonstrations, dangerous waterways) or to make safer food and drink consumption decisions.

AI could have an important role in supporting travel-related disease surveillance activities and could become a useful tool for deriving insights from big data collected by the ISTM GeoSentinel® Network. AI models have already been used at one of the authors’ institutions to improve the accuracy of dengue diagnosis^8^ and similar approaches could be used with other illnesses in returned travelers. Lin and Hou^9^ summarized efforts in Eastern Asia to deploy AI-based technology in the diagnosis and classification of COVID-19, targeting public health messages and enforcing home quarantine measures. Lessons learned for future pandemics will prove invaluable.

Data relating to travelers who travel abroad for planned healthcare remain sparse, rendering the counseling of medical tourists quite limited. Here again, AI may serve a useful role if it is embraced by medical tourism healthcare providers returning data to a central registry. Finally, future travelers and especially those with pre-existing conditions will benefit from real-time feedback from wearable or implanted biosensors or even nanotechnology tattoos which will provide timely reminders of important health interventions and alert them to detected deteriorations in their underlying illnesses. This could be useful for predicting hypoglycemic episodes in diabetic travelers or exacerbations of asthma during travel to polluted cities, for example.

Much of the excitement surrounding the transformative potential of AI resides in the medical research domain but applications are gradually being integrated into clinical practice. It is likely that we will witness extraordinary developments in this arena in the next decade. Travel medicine should position itself to become an early adopter of AI technology. We expect that AI will soon become a core component of undergraduate medical curricula. The ISTM and regional travel medicine societies and faculties play a key role in postgraduate travel health education. We should collaborate with data scientists and invite them to speak at our conferences and webinars. The ISTM body of knowledge should be updated to include AI and other technological advances in the field. Research grant funding calls should be issued to encourage interdisciplinary cooperation between AI experts and travel medicine researchers. Concerns about erosion of the clinician–patient relationship, loss of essential clinical skills, data privacy and the risks associated with biased data should be openly discussed.^10^ Travel medicine, as one of the newest areas of clinical practice and research with a truly global dimension, is uniquely positioned to harness the exciting benefits of AI.

## Funding

This research did not receive any specific grant from funding agencies in the public, commercial or not-for-profit sectors.

## Declaration of interests

The authors have no conflicts of interest to declare.
